# Equal Service, Equal Honour

**Published:** 1893-04-29

**Authors:** 


### The Hospital

An Institutional Journal of

Science, Medicine, Nursing, and Philanthropy.

*For Hospitals, Asylums, Infirmaries, Dispensaries, Medical Practitioners, Students, Nurses, and the Charitable Public.*

Vol. XIV.—No. 344.] April 29, 1893.

### Equal Service, Equal Honour.

We confess to a settled regard for monarchy, aristocracy, and everything which can give adornment and completeness to the outward symbolism of Imperial states. Democracy has its own peculiar merits; but its natural and necessary complement, not its antithesis, is aristocracy. Those democratic persons who can see a fitness in aristocracy—and they are many—always make it a condition that the aristocracy shall be true in substance, and, as far as possible, representative. High birth, it is admitted, may lay claim to distinction; so, too, in many cases. may great wealth. But we submit that on a full equality with these should be placed high intellectual capacity, noble achievement in science or literature, and great public services. These are truisms, and require no argumentation.

Ours are the days of democratic aristocracy, if the apparent paradox may be permitted. Nobody complained when Benjamin Disraeli was raised to the peerage. On the contrary, there was a universal shout of agreement from all sorts and conditions of men. If his great rival, Mr. Gladstone, had passed to the Lords after 1886, everybody, including the most democratic of his followers, would have considered that a fitting crown had been placed upon a long and distinguished career. It is a good thing to honour great service; it is a bad thing not to honour it.

It used to be said of the armies of the great Napoleon that every private soldier carried a field-marshal's baton in his knapsack. It may now be said, with one exception, of every honourable man in Britain that he carries a peerage in his pocket. The solitary exception is the doctor. By merely entering the medical profession a man casts away for ever his chances of all first-rate State honours. A knighthood he may attain to, and so may his grocer. Even a baronetcy is within his reach, as it is within the reach of a second-rate railway contractor. But that appears to be the doctor's limit. The railway contractor ascends by easy steps to the full honours of the peerage.

It cannot be expected that this state of things will satisfy medical men. They would be less than human if it did. The *British Medical Journal* has waked many a sympathetic echo by demanding a life peerage for Sir Joseph Fayrer. If Sir Joseph Fayrer's claims be considered inadequate, the medical profession may give up all hope. Sir Joseph was an army surgeon so long ago as 1848. He passed through the Indian Mutiny. At the defence of Lucknow Sir Henry Lawrence died in "Fayrer's house," and in its master's arms. Sir Joseph was the medical guide, philosopher, and friend of the Prince of Wales all through his Indian tour in 1876. Professional honours of all kinds have been heaped upon him in Britain, in Europe, and in America. He has been for many years president of the India Medical Board. In addition to all this, he is an accomplished naturalist, and has written, among other books, the standard work on the *Thanatophidia of India.* As we have said, if all these public services do not entitle Sir Joseph Fayrer to a life peerage, then doctors may make up their minds that England, at any rate, has not the knowledge or the culture necessary for the appreciation of scientific medicine.

It has been whispered that no Government dare make a medical man a peer on pain of rousing to fever pitch the slumbering jealousy of his professional rivals. That we cannot believe. Medical men, we know, are human, some of them very human. But as a class, they are not more human than other professional persons; than clergymen, for example; or than lawyers. The worthiest medical men of to-day feel the slight of unrecognized merit much more on behalf of their profession than they do for their own individual sakes. It does not really matter to the individual what the world thinks of him so long as he can, as Carlyle said, "get his life lived honestly," and make a modest provision for his old age and for his children's start in life. A rational man at seventy probably thinks very little of the peerage. We are sure that Sir Joseph Fayrer thinks nothing at all of it. But the younger men, the men of generous instincts, of professional loyalty, the men who are beginning to understand the kind of capacity and character which are required to carry a man through all the intermediate stages to the very front rank of the medical profession—these men know how entirely worthy of honour their distinguished seniors are; and they feel a great deal of very honourable bitterness when those seniors are passed by in favour of men who have done nothing for humanity, nothing for the State, but have only filled their own pockets at the cost of other men's toil.

Medical men neither threaten nor make a noise. If they did they would carry their point. But it is not good policy to give everything he asks to the sturdy beggar and to deny the useful servant his just reward. The last Prime Minister and the present are both men who have keen eyes for merit and capacity. Both of them have shown a knowledge of modern medicine and its elevating and expanding tendency which is much to their honour. They can give a great impulse to the wider, profounder, and more philosophic study of medicine by selecting the broad, deep, and philosophic representatives of it for special recognition and distinction. The want of equality in honour depresses men exactly in the same way as the want of any other equality. It begets a sense of injustice; it sours, even if but a little, the very finest of human grapes.

The younger generation of medical men have made up their minds that some of their seniors who are now living shall take their place among the men who are most honoured in the land, and that by indisputable right. It is not that these younger men are blinded by the glamour of the peerage. They are much too good physiologists for that. We know, and nothing can persuade us otherwise, that some of the leaders of medicine are among the most potent forces for good which the nineteenth century has produced. Their knowledge is immense, their honesty is like the leaven and the salt of the New Testament parables. They are singularly open in mind, and free from prejudice. They influence literature and life at a thousand points. Thanks very largely to them, human knowledge is becoming wider, deeper, and more philosophical with a rapidity unknown before. The British physicians, surgeons, teachers, and researchers of the present day exert the influence of a hundred Aristotles.

Services like these the country and the Government of the country cannot afford to ignore. Our aristocracy, to serve the highest purposes of an aristocracy, must, as we have said, be real and true in substance; and it must be representative of all classes. It must consist of the best of all classes. Then it will command the veneration and the influence which it ought to command, but not otherwise.

The exclusion of distinguished medical scientists from the peerage cannot be defended on any ground of expediency, still less on any ground of reason. It is a gratuitous insult to one of the noblest of the professions. We, who are among the loyallest of the Queen's subjects, feel that we are put upon a most distasteful and odious task, in that we are compelled from time to time to insist that our profession in the persons of its leaders shall be placed on an equality of honour with all the other classes of her Majesty's subjects. Literature, in the person of Lord Tennyson, added to the peerage one of its most distinguished ornaments. Science, in the person of Lord Kelvin, has made an illustrious body still more illustrious. We insist once more that the House of Peers can never be complete, or completely representative, until it is adorned by one or more of the many distinguished medical scientists, who confer honour upon our country and our generation.

